# Evaluation of HER2/neu Expression in Metastatic Axillary Lymph Node Tissue of Breast Cancer Patients Using [99mTc]Tc-(HE)3-G3

**DOI:** 10.32607/actanaturae.27448

**Published:** 2024

**Authors:** O. D. Bragina, L. A. Tashireva, D. M. Loos, S. V. Vtorushin, A. A. Shulga, E. N. Konovalova, M. E. Borodina, V. I. Chernov, V. M. Tolmachev, S. M. Deyev

**Affiliations:** Tomsk Cancer Research Institute, Tomsk, 634009 Russian Federation; National Research Tomsk Polytechnic University, Tomsk, 634050 Russian Federation; Siberian State Medical University, Tomsk, 634050 Russian Federation; Shemyakin–Ovchinnikov Institute of Bioorganic Chemistry, Moscow, 117997 Russian Federation; Hertsen Moscow Oncology Research Institute, Moscow, 125284 Russian Federation; National Research Center Kurchatov Institute, Moscow, 123098 Russian Federation; Uppsala University, Uppsala, 75185 Sweden

**Keywords:** breast cancer, lymph node metastasis, DARPinG3, HER2/neu, radionuclide diagnostics

## Abstract

Anatomic visualization and molecular typing of metastatic regional lymph nodes in breast cancer patients are a serious clinical challenge in modern oncology. According to the results of previous studies, [99mTc]Tc-(HE)3-G3 has proven to be a promising diagnostic agent in differentiating the HER2/neu receptor status in primary breast tumors (*p* < 0.05, Mann–Whitney test). In this regard, the purpose of this study is to explore the possibilities of using [99mTc]Tc-(HE)3-G3 to determine the HER2/neu receptor status in the metastatic axillary lymph nodes (mALNs) of breast cancer patients. The study was conducted using clinical material from 20 breast cancer patients (T2-4N1-3M0-1) before systemic therapy (10 patients with positive and 10 patients with negative HER2/neu expression in mALNs) who underwent SPECT/CT scan 4 h after the administration of [99mTc]Tc-(HE)3-G3. Morphological and immunohistochemical studies of mALNs with assessment of the HER2/neu status were performed on all patients. We found that mALN-to-background and mALN-to-latissimus dorsi muscle ratios for [99mTc]Tc-(HE)3-G3 uptake 4 h after its administration may be used for typing of the HER2/neu status in mALNs of breast cancer patients (*p* < 0.05, Mann–Whitney test). In that case, sensitivity and specificity for the mALN-to-background ratio were identical at 80%, with the threshold value being > 12.25.

## INTRODUCTION

The condition of regional lymph nodes in breast cancer (BC) is an important prognostic factor that is significant both in choosing the modality of local and systemic therapies for these patients and in assessing the prognosis of the disease [1]. Unfortunately, traditional diagnostic methods, such as ultrasound (US), mammography, magnetic resonance imaging, and computed tomography (CT), are not characterized by high sensitivity and specificity levels in differentiating normal and metastatic lymph node structures, which leads to a large number of false-positive and false-negative results in preclinical cancer staging [2, 3]. However, there is a need not only for anatomical detection, but also for assessing the molecular profile of all identified metastatic foci, which is an important factor in the evaluation of the tumor spread and the determination of indications for prescribing directed (targeted) therapy in BC patients, an approach that significantly improves overall and relapse-free survival rates [4, 5].

In recent years, there has been an active effort to investigate targeted radionuclidic imaging techniques that could help detect a specific molecular target [6, 7]. A particular example is the results of studies using alternative scaffold proteins that are labeled with various radioisotopes and targeted at human epidermal growth factor receptor-2 (HER2/neu) [8, 9]. These constructs offer optimal characteristics in delivering a diagnostic isotope to a target antigen: high specificity and affinity, low toxicity, and rapid elimination from the patient’s body, which significantly reduces the time from agent injection to the onset of a diagnostic procedure [10, 11, 12].

For example, the data of phase I clinical trials of the agents [99mTc]Tc-ADAPT6 (ClinicalTrials. gov Identifier: NCT03991260 and ClinicalTrials.gov Identifier: NCT05412446) and 99mTc-ZHER2:41071 (ClinicalTrials.gov Identifier: NCT05203497) performed at the Department of Radionuclide Therapy and Diagnostics of the Cancer Research Institute of the Tomsk National Research Medical Center (CRI TNRMC) demonstrated that it is possibile to determine HER2/neu status in the primary tumor [13, 14] and metastatic lymph nodes in BC patients [15]. Another agent promising for targeted radionuclide diagnosis of HER2-positive breast cancer is a designed ankyrin repeat protein (DARPinG3) molecule that is constructed on the basis of 14 to 21 kDa ankyrin proteins and exhibits a high tropism for epidermal growth factor receptor type 2 [16]. The data of preclinical *in vitro *studies of [^99m^Tc]Tc-(HE)_3_-G3 [17] demonstrated its rapid binding to the HER2/neu receptor and slow internalization in SKOV3 and BT-74 cell lines, as well as a higher uptake in HER2-positive SKOV3 xenografts compared with that in HER2-negative Ramos xenografts and a low liver uptake in *in vivo *studies. A phase I clinical trial of [^99m^Tc]Tc-(HE)_3_-G3 (ClinicalTrials.gov Identifier: NCT05695859) at a dose of 3,000 μg showed that it was safe for BC patients and highly specific in assessing the HER2/neu status in the primary tumor using SPECT without CT [18].

The purpose of this study was to investigate the possibility of the clinical use of the radiopharmaceutical [^99m^Tc]Tc-(HE)_3_-G3 in order to determine the HER2/neu status in the metastatic axillary lymph nodes of BC patients and identify optimal parameters for determining the receptor’s positive and negative status.

## EXPERIMENTAL

**Protein production**

DARPin(HE)3-G3 (amino acid sequence: MRGSHEHEHEGSDLGKKLLEAARAGQDDEVRILMANGADVNAKEYGLTPYLATAHGHLEIVEVLLKNGADVNAVDAIGFTPLHLAAFIGHLEIAEVLLKH GADVNAQDKFGKTAFDISIGNGNEDLAEILQKLN) was synthesized at the Institute of Biological Chemistry.

**Characterization of clinical material**

This was an open, non-randomized, and prospective study that started after registration at ClinicalTrials. gov (Identifier: NCT15122022), approval by the bioethical committee of CRI TNRMC, and the completion of an informed consent form by patients before administration of the radiopharmaceutical. The study included 20 BC patients with metastatic axillary lymph nodes (mALNs) (T2-4N1-3M0-1) before the start of systemic or local treatment. Human epidermal growth factor receptor HER2/neu expression in mALNs was positive in 10 patients (*n *= 10) and negative in 10 patients (*n *= 10). The mean age of the patients included in the study was 49.6 years.

At the preclinical stage, all patients underwent a comprehensive clinical and instrumental examination according to the 2023 Russian Society of Clinical Oncology (RUSSCO) protocols. The presence, anatomical location, and size of tumor nodes in the mammary gland and axillary region were determined using US. The mean primary tumor size was 24 ± 5 mm, and the mean metastatic axillary lymph node size was 20 ± 3 mm.

**Morphological and immunohistochemical studies**

In all the cases, morphological and immunohistochemical (IHC) studies of the biopsy and/or surgical material of metastatic axillary nodes were performed to determine the HER2/neu status of the largest lymph node using standard methods. The surgical material of patients who had started treatment directly from the surgical stage was studied. Metastatic lymph nodes were marked for IHC analysis under US guidance by placing a localization mark before surgical treatment. HER2/neu expression with IHC 3+ or IHC 2+ and a positive FISH (fluorescence *in situ *hybridization) was considered positive, and that with IHC 0 or 1+ was considered negative, which corresponded to the 2018 ASCO/CAP (American Society of Clinical Oncology and College of American Pathologists) criteria [19, 20]. IHC was a reference method, and its data were compared with data from the radionuclide analysis.

**Preparation of the radiopharmaceutical**

The radiopharmaceutical [^99m^Tc]Tc-(HE)_3_-G3 in a dose of 3,000 μg was prepared immediately before intravenous administration to patients at the Department of Radionuclide Therapy and Diagnostics of CRI TNRMC using the protocol described previously [18]. [^99m^Tc]Tc-(HE)_3_-G3 was purified by size-exclusion chromatography using sterilized NAP-5 columns (Sephadex G-25, GE, Healthcare, Chicago, IL, USA) pre-equilibrated and eluted with a sterile sodium phosphate buffer. The purified fraction was brought to a volume of 10 mL using a sterile isotonic NaCl solution. A 2 μL aliquot of the compound solution was used for pH determination and radiochemical purity analysis. The pH of the radiopharmaceutical solutions was determined using pH test strips. Radiochemical purity was analyzed using instant thin layer chromatography (Agilent Technologies, Santa Clara, CA, USA).

**Radionuclide study protocol**

[^99m^Tc]Tc-(HE)_3_-G3 uptake was assessed by measuring the maximum standardized uptake (SUVmax) in mALNs, the projections of contralateral axillary lymph nodes, and those of reference organs, such as the liver, latissimus dorsi, and spleen 4 h after its administration. Additionally, parameters such as mALN-to-background and mALN-to-reference organs were calculated for each patient (*Table 1*). SUV_max_ was determined in the largest mALN based on the anatomical location corresponding to the US description and biopsy sampling.

**Table 1:** [99mTc]Tc-(HE)3-G3 uptake in mALNs (SUV_max_) and reference organs and mALN-to-reference organ ratios in BC patients

	SUV_max_ (mALN)	SUV_max_ (background mALN)	mALN/ background	SUV_max_ (liver)	SUV_max_ (LDM)	SUV_max_ (spleen)	mALN/ liver	mALN/ LDM	mALN/ spleen
HER2-positive mALNs										
1	1.8	0.3	6.7	9.1	0.3	4.0	0.2	6.2	0.5
2	2.6	0.2	15.2	5.2	0.3	2.5	0.5	8.6	1.04
3	2.2	0.2	13.5	3.0	0.3	1.3	0.7	6.2	1.7
4	10.7	0.3	33.3	4.7	0.4	2.5	2.3	26.0	4.3
5	8.7	0.3	34.9	5.7	0.4	2.1	1.5	21.3	4.2
6	2.4	0.4	5.9	4.1	0.2	1.7	0.6	10.9	1.5
7	14.0	0.3	41.2	2.9	0.5	3.1	4.9	25.9	4.5
8	6.5	0.1	50.3	8.7	0.4	4.2	0.8	17.7	1.6
18	8.7	0.4	23.5	3.4	0.3	4.4	2.6	27.2	1.9
19	4.8	0.1	36.9	6.9	0.3	0.1	0.7	15.0	4.8
	6.2 ± 4.2	0.3 ± 1.1	26.1 ± 15.4	5.4 ± 2.2	0.34 ± 0.1	2.6 ± 1.4	1.5 ± 1.4	16.5 ± 8.3	2.6 ± 1.6
HER2-negative mALNs										
9	3.9	0.5	8.6	6.3	0.5	2.1	0.6	8.4	1.8
10	3.1	0.4	8.5	15.2	0.2	8.1	0.2	21.1	0.3
11	1.2	0.1	11.0	0.6	0.3	4.9	2.2	4.5	0.2
12	0.5	0.2	2.3	2.7	0.0	0.4	0.2	13.2	1.3
13	3.8	0.3	13.7	9.7	0.7	5.6	0.4	5.2	0.7
14	6.8	0.4	18.9	6.2	0.6	1.9	1.1	11.4	3.5
15	6.8	0.7	10.4	10.3	0.8	3.7	0.7	8.7	1.8
16	1.0	0.7	1.5	13.8	0.5	6.6	0.1	2.1	0.1
17	5.6	0.5	10.8	10.1	0.6	2.5	0.6	9.5	2.3
20	1.7	0.4	4.5	1.5	0.3	0.9	1.2	5.6	1.8
	3.4 ± 2.4	0.4 ± 0.2	9.0 ± 5.3	7.6 ± 5.0	0.5 ± 0.2	3.7 ± 2.6	0.7 ± 0.6	8.9 ± 5.4	1.4 ± 1.1

Note: mALN is a metastatic axillary lymph node; LMS is the latissimus dorsi muscle.

Radionuclide studies in BC patients 4 h after administration were performed on a Siemens Symbia Intevo Bold gamma camera equipped with a lowpower and high-resolution collimator. In all cases, SPECT/CT of the chest and upper abdomen with reconstruction was performed using the xSPECT protocol (Siemens). The images were processed using the Syngo.via software (Siemens).

**Statistical methods**

Data were analyzed and visualized using the Prism 10 software (GraphPad). Values are presented as a mean ± standard deviation (M ± SD) or median and interquartile range (Me(Q1–3)). The differences in organ uptake at different time points were analyzed using the one-way analysis of variance (ANOVA). The nonparametric Mann–hitney test was used to evaluate the significance of the differences between the parameters of HER2-positive and HER2-negative tumors. ROC analysis was performed to evaluate the predictive values of the parameters. All criteria were two-sided, and the differences were considered significant at *p* < 0.05.

## RESULTS

**IHC studies**

The immunohistochemical analysis revealed that the HER2/neu receptor status was identical in the primary tumors and mALNs of all BC patients included in the study.

**[^99m^Tc]Tc-(HE)_3_-G3 labeling and radionuclidic studies**

**Fig. 1:**
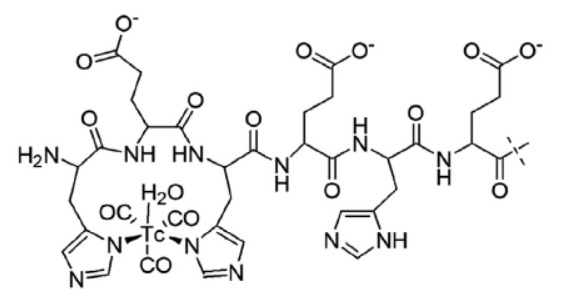
Schematic of the labeling of technetium-99m with a DARPinG3 molecule using the tricarbonyl technique

Labeling of the radiopharmaceutical (*Fig. 1*) and radionuclidic imaging in all BC patients included in the study were performed according to the protocols described in the Experimental section. The radiochemical purity of [^99m^Tc]Tc-(HE)_3_-G3 was 98.7 ± 1.8%. The mean administered dose activity was 435 ± 138 MBq.

**[^99m^Tc]Tc-(HE)_3_-G3 uptake in metastatic and contralateral axillary lymph nodes**

**Fig. 2:**
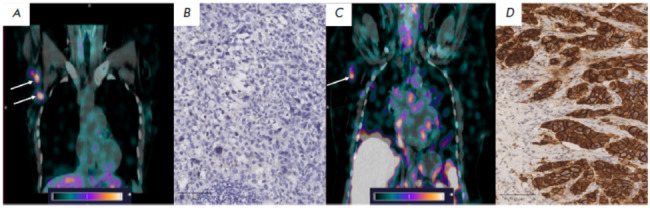
[^99m^Tc]Tc-(HE)_3_-G3 uptake in mALNs of BC patients 4 h after administration: (*A*) – [^99m^Tc]Tc-(HE)_3_-G3 uptake in HER2-positive mALNs (indicated by white arrows); (*B*) – IHC imaging of a HER2-positive mALN (×400); (*C*) – [^99m^Tc]Tc-(HE)_3_-G3 uptake in a HER2-negative mALN (indicated by the white arrow); (*D*) – IHC imaging of a HER2-negative mALN (×400)

mALNs were visualized in all BC patients, regardless of the HER2/neu status (*Fig. 2*). Quantitative data on [^99m^Tc]Tc-(HE)_3_-G3 uptake in anatomical structures are shown in *Table 1*.

**Fig. 3:**
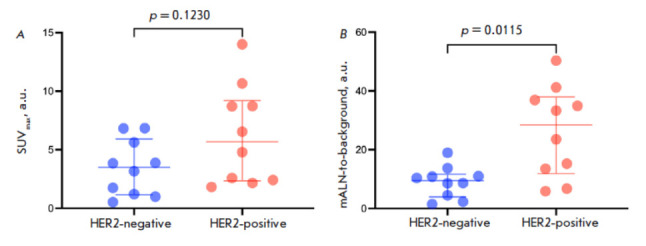
SUVmax (*A*) and mALN-to-background ratio (*B*) 4 h after administration of [^99m^Tc]Tc-(HE)_3_-G3 to BC patients with HER2-positive and HER2-negative mALNs

There were no differences in SUVmax among BC patients with different HER2/neu statuses in mALNs (6.2 ± 4.2 for positive expression and 3.4 ± 2.4 for negative expression) (*p *= 0.1230, Mann–Whitney test). However, there were statistical differences in the mALN-to-background ratios: it was higher in the subgroup of patients with a HER2-positive mALN status (26.1 ± 15.4) than in the subgroup with a HER2-negative mALN status (9.0 ± 5.3) (*p* = 0.0115, Mann–Whitney test) (*Table 1*, *Fig. 3*).

**[^99m^Tc]Tc-(HE)_3_-G3 uptake in reference organs and mALN-to-reference organ ratio**

The SUVmax of [^99m^Tc]Tc-(HE)_3_-G3 in the liver, LDM, and spleen was 5.4 ± 2.2, 0.4 ± 0.1, and 2.6 ± 1.4 and 7.6 ± 5.0, 0.5 ± 0.2, and 3.7 ± 2.6 for HER2-positive and HER2-negative mALNs, respectively. There were no statistical differences in the [^99m^Tc]Tc-(HE)_3_-G3 uptake in each organ for the positive and negative HER2/neu statuses (*p *> 0.05, Mann–Whitney test).

**Fig. 4:**
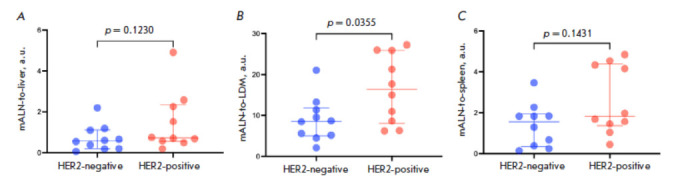
mALN-to-liver (*A*), mALN-to-LDM (*B*), and mALN-to-spleen ratios (*C*) 4 h after administration of [^99m^Tc]Tc-(HE)_3_-G3

Calculations of mALN-to-reference organ ratios revealed that the mALN-to-LDM ratio was higher in HER2-positive mALNs than in HER2-negative mALNs (16.5 ± 8.3 and 8.9 ± 5.4, respectively) (*p* = 0.035, Mann–Whitney test) (*Table 1*, *Fig. 4*).

**Determining the most informative parameter in assessing the HER2/neu status in mALNs of BC patients using [^99m^Tc]Tc-(HE)_3_-G3**

**Fig. 5:**
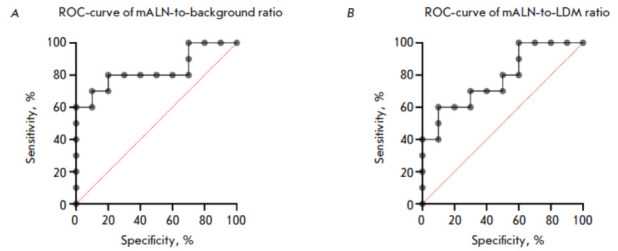
ROC-curves of mALN-to-background (*A*) and mALN-to-LDM (*B*) ratios upon HER2/neu status detection in mALNs of BC patients 4 h after administration of [^99m^Tc]Tc-(HE)_3_-G3

The most informative parameter for assessing the HER2/neu status in mALNs using [^99m^Tc]Tc-(HE)_3_-G3 was determined by ROC analysis that identified the sensitivity and specificity parameters for each of them. The most sensitive and specific parameter for determining the HER2/neu status in the mALNs of BC patients using [^99m^Tc]Tc-(HE)_3_-G3 was the mALN-to-background ratio: AUC of 0.83 (95% CI 0.63–1.00), sensitivity of 80%, and specificity of 80%; a threshold value of > 12.25 a.u. For the mALN-to-LDM ratio, these parameters were as follows: AUC of 0.78 (95% CI 0.58–1.00), sensitivity of 70%, and specificity of 70%; a threshold value of > 10.25 a.u. (*Fig. 5*).

## DISCUSSION

The use of alternative scaffold proteins for radionuclidic receptor imaging of malignant tumors has been one of the promising developments in the field over the last 10 years. This is primarily due to the high specificity of the targeted delivery molecules and the shorter time interval between agent administration and the start of examination. Furthermore, the conduct of the diagnostic stage using modern devices that combine positron emission tomography and single- photon emission computed tomography with CT data provides a more accurate anatomical visualization and measurement of the administered agent’s uptake *in vivo*.

Phase I clinical trials conducted at the Department of Radionuclide Therapy and Diagnostics of CRI TNRMC on HER2/neu in BC patients using a number of diagnostic radiopharmaceuticals ([^99m^Tc]Tc-(HE)_3_-G3, [99mTc]Tc-ADAPT6, and 99mTc-ZHER2:41071) [13, 14, 18] have demonstrated not only the safe character of the procedure, but also the possibility of typing primary breast tumors depending on their HER2/neu status (*p* < 0.05, Mann– Whitney test) [21]. These findings and expansion of research towards locally advanced and metastatic BC forms promoted the planning and initiation of phase II clinical trials using [99mTc]Tc-ADAPT6 and [^99m^Tc]Tc-(HE)_3_-G3.

Previously published data on the use of the radiopharmaceutical [99mTc]Tc-ADAPT6 to determine the HER2/neu status in nALNs of BC patients demonstrated its high uptake (SUVmax = 8.7 ± 4.6) and a significant difference between HER2-positive and HER2-negative foci (*p* < 0.05, Mann–Whitney test). The ROC analysis revealed that using the threshold SUVmax value (4.22) in mALNs provides a 92% sensitivity level and 100% specificity [15].

In the present study, the highest statistical differences between HER2-positive and HER2-negative mALNs in BC patients 4 h after the administration of [^99m^Tc]Tc-(HE)_3_-G3 were observed for a mALN-tobackground ratio of 26.1 ± 15.4 (*p *= 0.0115, Mann– Whitney test). According to the ROC analysis, the threshold value of the mALN-to-background ratio was 12.25, and sensitivity and specificity stood at an identical 80%.

These findings partially confirm previously reported data from preclinical and clinical trials of a comparative analysis of the diagnostic efficacy of [99mTc]Tc-ADAPT6 and [^99m^Tc]Tc-(HE)_3_-G3 [22]. For example, sequential administration of both diagnostic agents at an interval of 3 days before the start of systemic therapy in 11 HER2-positive BC patients demonstrated a higher uptake of [99mTc]Tc-ADAPT6 by primary breast tumors (SUVmax = 4.7 ± 2.1) 2 h after administration compared with that of [^99m^Tc]Tc-(HE)_3_-G3 (SUVmax = 3.5 ± 1.7) 4 h after administration (*p* < 0.005, paired *t*-test). In this case, the tumor-tobackground ratio was not statistically different for both agents (15.2 ± 7.4 for [99mTc]Tc-ADAPT6 and 19.6 ± 12.4 for [^99m^Tc]Tc-(HE)_3_-G3) (*p *> 0.05, paired* t*-test) [23].

According to both studies, [99mTc]Tc-ADAPT6 proved to be the optimal agent for the typing of primary breast tumors, which provides the opportunity to differentiate the HER2/neu receptor status. This is important for optimizing the diagnostic stage and prescribing targeted therapy.

Given that, unlike the ADAPT6 protein, [^99m^Tc]Tc-(HE)_3_-G3 does not compete with trastuzumab because it binds to other HER2/neu epitopes, and the radiopharmaceutical may be useful in clinical practice to evaluate the monitoring of preoperative systemic therapy in patients with HER2/neu overexpression.

## CONCLUSIONS

[^99m^Tc]Tc-(HE)_3_-G3 proved effective in differentiating the HER2/neu status in the mALNs of BC patients and demonstrated mALN-to-background ratios with 80% sensitivity and 80% specificity. To expand the indications for clinical use, [^99m^Tc]Tc-(HE)_3_-G3 should be further studied in the dynamics of preoperative systemic therapy in BC patients with HER2/neu overexpression.

## Abbreviations

BC: breast cancer
US: ultrasound
CT: computed tomography
HER2/neu: human epidermal growth factor receptor-2
RP: radiopharmaceutical
mALN: metastatic axillary lymph node
IHC: immunohistochemistry
FISH: fluorescence in situ hybridization
ASCO/CAP: American Society of Clinical Oncology and College of American Pathologists
SPECT: single-photon emission computed tomography
LDM: latissimus dorsi muscle
SUV: standardized uptake value
